# A low-temperature phase of the 1:1 complex of 2-(6-diethylamino-3-diethyl­iminio-3*H*-xanthen-9-yl)benzoate with ethyl gallate at 93 K

**DOI:** 10.1107/S1600536808016528

**Published:** 2008-06-07

**Authors:** Jin Mizuguchi

**Affiliations:** a Department of Applied Physics, Graduate School of Engineering, Yokohama National University, 79-5 Tokiwadai, Hodogaya-ku, 240-8501 Yokohama, Japan

## Abstract

The title compound, C_28_H_30_N_2_O_3_·C_9_H_10_O_5_, is a well known red leuco complex of 2-(6-diethylamino-3-diethyl­iminio-3*H*-xanthen-9-yl)benzoate (rhodamine B base abbreviated to RBB, a leuco dye) with ethyl gallate (EG, developer). The structure of the complex at room temperature has recently been reported by Sekiguchi, Takayama, Gotanda & Sano [*Chem. Lett.* (2007 ▶), **36**, 1010–1011]. We have found a new phase of the material with two discrete base/developer complexes (RBB-A/EG-A and RBB-B/EG-B) in the asymmetric unit at 93 K. There are no significant differences between the two developer mol­ecules EG-A and EG-B. The lactone ring of RBB is opened in each mol­ecule to form a zwitterionic structure. However, the xanthene system is almost flat in RBB-A (r.m.s. deviation 0.0234 Å) but is less so in RBB-B (r.m.s. deviation 0.1095 Å). Furthermore, the ethyl groups of the xanthene diethyl­amino substituents lie on the same side of the xanthene plane in RBB-A but on opposite sides in RBB-B. Dimeric dye/developer complexes are formed through inter- and intra­molecular O—H⋯O hydrogen bonds and are linked further into dimers by additional O—H⋯O hydrogen bonds involving either EG-A or EG-B developer mol­ecules.

## Related literature

For general background literature on leuco dyes, see: Muthyala (1997 ▶). For the structure of the 1:1 RBB/EG complex at room temperature, see: Sekiguchi *et al.* (2007 ▶). For the related structure of *n*-propyl gallate, see: Iwata *et al.* (2005 ▶); Hitachi *et al.* (2005 ▶); Mizuguchi *et al.* (2005 ▶).
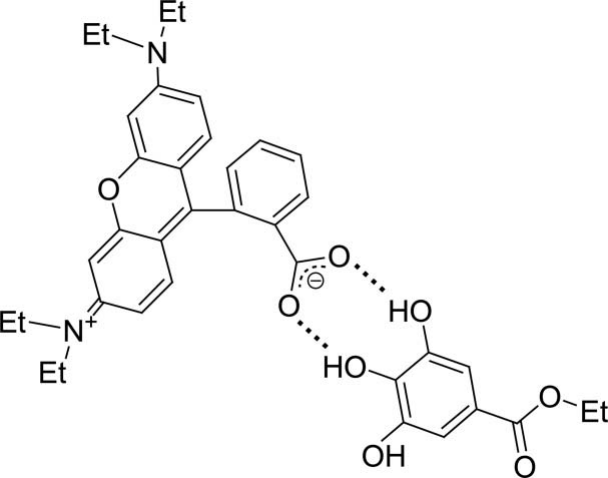


## Experimental

### 

#### Crystal data

C_28_H_30_N_2_O_3_·C_9_H_10_O_5_
*M* *_r_* = 640.71
Triclinic, 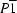

*a* = 11.3689 (2) Å
*b* = 16.3654 (3) Å
*c* = 17.6518 (3) Å
α = 94.1760 (7)°
β = 96.1440 (7)°
γ = 93.7790 (7)°
*V* = 3247.69 (10) Å^3^
*Z* = 4
Cu *K*α radiation
μ = 0.76 mm^−1^
*T* = 93 K
0.20 × 0.20 × 0.20 mm

#### Data collection

Rigaku R-AXIS RAPID diffractometer
Absorption correction: multi-scan (Higashi, 1995 ▶) *T*_min_ = 0.810, *T*_max_ = 0.860
29633 measured reflections
11011 independent reflections
7318 reflections with *F* ^2^ > 2σ(*F* ^2^)
*R*_int_ = 0.039

#### Refinement

*R*[*F* ^2^ > 2σ(*F* ^2^)] = 0.044
*wR*(*F* ^2^) = 0.115
*S* = 0.94
11011 reflections
881 parameters
H atoms treated by a mixture of independent and constrained refinement
Δρ_max_ = 0.23 e Å^−3^
Δρ_min_ = −0.25 e Å^−3^

### 

Data collection: *PROCESS-AUTO* (Rigaku, 1998 ▶); cell refinement: *PROCESS-AUTO*; data reduction: *CrystalStructure* (Rigaku/MSC, 2006 ▶); program(s) used to solve structure: *SIR2004* (Burla *et al.*, 2005 ▶); program(s) used to refine structure: *SHELXL97* (Sheldrick, 2008 ▶); molecular graphics: *ORTEPIII* (Burnett & Johnson, 1996 ▶); software used to prepare material for publication: *CrystalStructure*.

## Supplementary Material

Crystal structure: contains datablocks General, I. DOI: 10.1107/S1600536808016528/sj2498sup1.cif

Structure factors: contains datablocks I. DOI: 10.1107/S1600536808016528/sj2498Isup2.hkl

Additional supplementary materials:  crystallographic information; 3D view; checkCIF report

## Figures and Tables

**Table 1:** Hydrogen-bond geometry (Å, °)

*D*—H⋯*A*	*D*—H	H⋯*A*	*D*⋯*A*	*D*—H⋯*A*
O6*A*—H6*A*O⋯O2*A*	0.94 (2)	2.72 (2)	3.4413 (18)	133.8 (18)
O6*A*—H6*A*O⋯O3*A*	0.94 (2)	1.72 (2)	2.6303 (17)	162 (2)
O6*A*—H6*A*O⋯O5*A*	0.94 (2)	2.53 (2)	2.8733 (19)	102.2 (17)
O5*A*—H5*A*O⋯O2*A*	0.92 (2)	1.66 (2)	2.5690 (19)	167 (2)
O5*A*—H5*A*O⋯O3*A*	0.92 (2)	2.62 (2)	3.1884 (18)	120.4 (18)
O4*A*—H4*A*O⋯O7*A*^i^	0.92 (2)	1.86 (2)	2.779 (2)	178.8 (10)
O6*B*—H6*B*O⋯O2*B*	0.94 (2)	2.72 (2)	3.4156 (18)	131.4 (16)
O6*B*—H6*B*O⋯O3*B*	0.94 (2)	1.68 (2)	2.5811 (17)	159 (2)
O6*B*—H6*B*O⋯O5*B*	0.94 (2)	2.55 (2)	2.8799 (19)	101.0 (15)
O5*B*—H5*B*O⋯O2*B*	0.94 (2)	1.64 (2)	2.5636 (18)	166 (2)
O5*B*—H5*B*O⋯O3*B*	0.94 (2)	2.66 (2)	3.2473 (18)	121.5 (19)
O4*B*—H4*B*O⋯O7*B*^ii^	0.97 (2)	1.83 (2)	2.7957 (19)	175 (2)

Symmetry codes: (i) 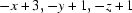; (ii) 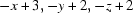.

**Fig. 1.**
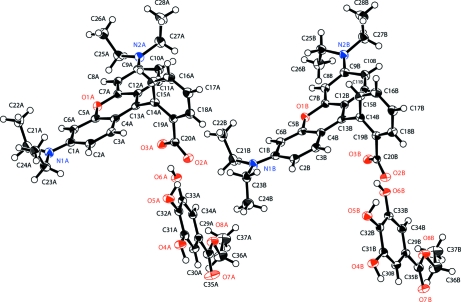
The structure of (I), showing 50% displacement ellipsoids and the atom numbering scheme.

**Fig. 2.**
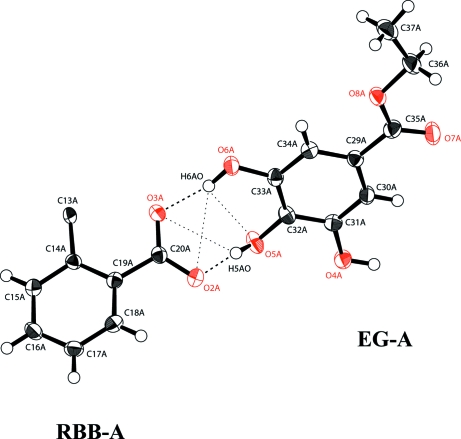
O—H···O intra- and intermolecular hydrogen bonds leading to the formation of the RBB-A/EG-A complex. For clarity only the C29A···C34A benzene ring and its substituents are shown for the RBB-A unit.

**Fig. 3.**
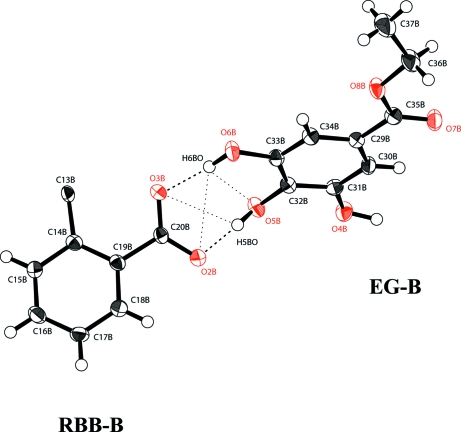
O—H···O intra- and intermolecular hydrogen bonds leading to the formation of the RBB-B/EG-B complex. For clarity only the C29B···C34B benzene ring and its substituents are shown for the RBB-B unit.

**Fig. 4.**
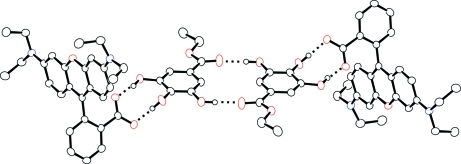
Hydrogen-bonded RBB-A···EG-A···EG-A···RBB-A dimers formed by linking two RBB/EG complexes.

## supplementary crystallographic information

### Comment

The coloration of colorless leuco dyes by reaction with acidic developers is well known (Muthyala, 1997) and is used in practice in thermal or rewritable papers. The coloration is usually interpreted as arising from the opening of the lactone ring due to proton transfer from the developer, acting as a proton donor, to the leuco dye, a proton acceptor.  The title compound [C_28_H_30_N_2_O_3_, C_9_H_10_N_2_O_5_] is a typical example of a leuco coloration system composed of 2-(6-diethylamino-3-diethyliminio-3H-xanthen-9-yl)benzoate [rhodamine B base (RBB): a leuco dye] with ethyl gallate (EG: a developer). The structure of the 1:1 dye:developer complex at room temperature has recently been reported by Sekiguchi *et al.* (2007). They found a dimer structure, in which two RBBs are connected by a sub dimer of EG through OH···O intermolecular hydrogen bonds. We have found a new phase of the material at 93 K with two discrete base/developer complexes RBB-A/EG-A and RBB-B/EG-B in the asymmetric unit, Fig. 1.

The lactone rings of each RBB are opened to form zwitterionic structures and the benzene rings with the anionic carboxylate substituents are twisted to be nearly perpendicular to the xanthene planes with dihedral angles: 84.68 (7)°) between the O1A/C4A/C5A/C7A/C12A/C13A and  C14A-C19A planes of RBB-A and 73.09 (7)° between the O1B/C4B/C5B/C7B/C12B/C13B and C14B-C19B planes of RBB-B. There are no significant differences between the two developer molecules EG-A and EG-B. The xanthene moiety is nearly flat in RBB-A (deviation from the least-squares plane, 0.0234Å) and the ethyl groups of the diethylamino substituents at each extremity of the xanthene lie on the same side of the xanthene plane. On the other hand, in RBB-B, the xanthene moiety deviates slightly from planarity by 0.1095Å and the ethyl groups of the diethylamino substituents lie on opposite sides of the xanthene plane. By comparison, the room temperature phase (Sekiguchi *et al.*, 2007) crystallises with  only one type of the molecule (eqivalent to RBB-A) in the asymmetric unit and with the ethyl groups of the diethylamino substituents all on the same side of the xanthene plane.

The dye/developer complexes are formed through intermolecular O5—H5O···O2, O6—H6O···O2, O5—H5O···O3 and O6—H6O···O3 hydrogen bonds with the planar configuration of this section of the molecule supported by intramolecular O6—H6O···O5 interactions, Figs. 2 & 3. In addition, O4—H4···O7 hydrogen-bonds form centrosymmetric RBB-A···EG-A···EG-A···RBB-A (Fig. 4), and RBB-B···EG-B···EG-B···RBB-B dimers. These are similar to those found in *n*-propyl gallate (Iwata *et al.*,  2005; Hitachi *et al.*,  2005; Mizuguchi *et al.*,  2005).

### Experimental

Rhodamine B base and 4-hydroxybenzophenone were purchased from Sigma-Aldrich Corp. and and Wako Pure Chemical Industries, Ltd., respectively. Single crystals of (I) were grown by recrystallization from a toluene solution which included an equimolar quantity of both chemicals. After 24 h, a number of red crystals were obtained in the form of blocks.

### Refinement

H4AO, H4BO, H5AO, H5BO, H6AO, and H6BO were located in electron density maps and were refined with isotropic displacement parameters. All the rest of the H atoms were placed in geometrically idealized position and constrained to ride on their parent atoms, with C—H = 0.93, 0.96, and 0.97 Å, and *U*_iso_(H) = 1.2 and 1.5 *U*_eq_(C), respectively.

### Crystal data

**Table:**

C_28_H_30_N_2_O_3_·C_9_H_10_O_5_	*Z* = 4
*M**_r_* = 640.71	*F*_000_ = 1360.00
Triclinic, *P*1	*D*_x_ = 1.310 Mg m^−^^3^
Hall symbol: -P 1	Cu *K*α radiation λ = 1.54187 Å
*a* = 11.3689 (2) Å	Cell parameters from 23114 reflections
*b* = 16.3654 (3) Å	θ = 3.0–68.5º
*c* = 17.6518 (3) Å	µ = 0.76 mm^−^^1^
α = 94.1760 (7)º	*T* = 93 K
β = 96.1440 (7)º	Block, red
γ = 93.7790 (7)º	0.20 × 0.20 × 0.20 mm
*V* = 3247.69 (10) Å^3^	

### Data collection

**Table:**

Rigaku R-AXIS RAPID diffractometer	7318 reflections with *F*^2^ > 2σ(*F*^2^)
Detector resolution: 10.00 pixels mm^-1^	*R*_int_ = 0.039
ω scans	θ_max_ = 68.3º
Absorption correction: multi-scan(Higashi, 1995)	*h* = −13→13
*T*_min_ = 0.810, *T*_max_ = 0.860	*k* = −19→19
29633 measured reflections	*l* = −19→19
11011 independent reflections	

### Refinement

**Table:**

Refinement on *F*^2^	H atoms treated by a mixture of independent and constrained refinement
*R*[*F*^2^ > 2σ(*F*^2^)] = 0.044	*w* = 1/[σ^2^(*F*_o_^2^) + (0.0668*P*)^2^] where *P* = (*F*_o_^2^ + 2*F*_c_^2^)/3
*wR*(*F*^2^) = 0.115	(Δ/σ)_max_ = 0.001
*S* = 0.94	Δρ_max_ = 0.23 e Å^−^^3^
11011 reflections	Δρ_min_ = −0.25 e Å^−^^3^
881 parameters	Extinction correction: none

### Special details

**Table:**

Geometry. All e.s.d.'s (except the e.s.d. in the dihedral angle between two l.s. planes) are estimated using the full covariance matrix. The cell e.s.d.'s are taken into account individually in the estimation of e.s.d.'s in distances, angles and torsion angles; correlations between e.s.d.'s in cell parameters are only used when they are defined by crystal symmetry. An approximate (isotropic) treatment of cell e.s.d.'s is used for estimating e.s.d.'s involving l.s. planes.
Refinement. Refinement of *F*^2^ against ALL reflections. The weighted *R*-factor *wR* and goodness of fit *S* are based on *F*^2^, conventional *R*-factors *R* are based on *F*, with *F* set to zero for negative *F*^2^. The threshold expression of *F*^2^ > σ(*F*^2^) is used only for calculating *R*-factors(gt) *etc*. and is not relevant to the choice of reflections for refinement. *R*-factors based on *F*^2^ are statistically about twice as large as those based on *F*, and *R*- factors based on ALL data will be even larger.

### Fractional atomic coordinates and isotropic or equivalent isotropic displacement parameters (Å^2^)

**Table:**

	*x*	*y*	*z*	*U*_iso_*/*U*_eq_	
O1B	0.87836 (10)	0.98739 (7)	0.42995 (7)	0.0229 (3)	
O2B	0.90792 (11)	0.95942 (8)	0.79262 (7)	0.0281 (3)	
O3B	0.93454 (11)	1.01742 (8)	0.68439 (7)	0.0267 (3)	
O1A	0.88374 (11)	0.47147 (7)	−0.06554 (7)	0.0240 (3)	
O2A	0.89121 (11)	0.48936 (8)	0.30288 (7)	0.0297 (3)	
O3A	0.92734 (11)	0.51496 (8)	0.18428 (7)	0.0293 (3)	
O6A	1.13694 (12)	0.58483 (8)	0.23956 (8)	0.0291 (3)	
O5A	1.09716 (13)	0.43281 (8)	0.30792 (8)	0.0338 (3)	
O4A	1.25444 (12)	0.39351 (8)	0.42047 (8)	0.0324 (3)	
O7A	1.57886 (13)	0.62783 (9)	0.45684 (9)	0.0469 (4)	
O8A	1.50522 (11)	0.71810 (8)	0.37821 (8)	0.0317 (3)	
O6B	1.13331 (12)	1.08929 (8)	0.74789 (8)	0.0288 (3)	
O5B	1.12346 (12)	0.92278 (8)	0.79090 (8)	0.0290 (3)	
O4B	1.29851 (12)	0.87659 (8)	0.88819 (8)	0.0302 (3)	
O7B	1.56742 (12)	1.13800 (8)	0.97180 (8)	0.0333 (3)	
O8B	1.48988 (11)	1.22678 (8)	0.89288 (8)	0.0322 (3)	
N2B	0.71903 (14)	1.23884 (9)	0.37466 (9)	0.0277 (4)	
N1B	0.97157 (14)	0.71094 (9)	0.43325 (9)	0.0274 (4)	
N2A	0.81195 (14)	0.74802 (9)	−0.10608 (9)	0.0268 (3)	
N1A	0.95787 (14)	0.19150 (9)	−0.04933 (9)	0.0288 (4)	
C9B	0.74323 (16)	1.18196 (11)	0.42495 (11)	0.0256 (4)	
C8B	0.80925 (16)	1.11476 (11)	0.40636 (11)	0.0240 (4)	
C7B	0.81752 (16)	1.05203 (11)	0.45422 (11)	0.0219 (4)	
C12B	0.76375 (16)	1.05087 (11)	0.52263 (11)	0.0219 (4)	
C11B	0.70771 (16)	1.12247 (11)	0.54336 (11)	0.0257 (4)	
C10B	0.69800 (17)	1.18574 (12)	0.49776 (11)	0.0280 (4)	
C13B	0.76693 (16)	0.98015 (11)	0.56424 (11)	0.0220 (4)	
C4B	0.82581 (15)	0.91354 (11)	0.53678 (11)	0.0214 (4)	
C5B	0.88086 (16)	0.91770 (11)	0.46921 (11)	0.0215 (4)	
C6B	0.93269 (16)	0.85388 (11)	0.43505 (11)	0.0237 (4)	
C1B	0.93259 (16)	0.77761 (11)	0.46880 (11)	0.0247 (4)	
C2B	0.88574 (16)	0.77399 (11)	0.54089 (11)	0.0252 (4)	
C3B	0.83370 (16)	0.83817 (11)	0.57228 (11)	0.0242 (4)	
C14B	0.69254 (16)	0.97331 (10)	0.62840 (11)	0.0222 (4)	
C15B	0.56987 (16)	0.96165 (11)	0.60911 (11)	0.0268 (4)	
C16B	0.49399 (17)	0.95451 (11)	0.66448 (12)	0.0298 (5)	
C17B	0.53913 (17)	0.95787 (11)	0.74086 (12)	0.0282 (4)	
C18B	0.66080 (16)	0.96716 (11)	0.76036 (12)	0.0270 (4)	
C19B	0.73859 (16)	0.97497 (11)	0.70563 (11)	0.0221 (4)	
C20B	0.87033 (16)	0.98517 (11)	0.72922 (11)	0.0213 (4)	
C27B	0.64851 (18)	1.30864 (12)	0.39121 (12)	0.0353 (5)	
C28B	0.51632 (19)	1.28710 (14)	0.36980 (13)	0.0500 (6)	
C25B	0.74973 (17)	1.22856 (12)	0.29529 (11)	0.0308 (5)	
C26B	0.87317 (18)	1.26447 (12)	0.28673 (12)	0.0359 (5)	
C23B	0.96527 (18)	0.62858 (11)	0.46230 (12)	0.0311 (5)	
C24B	1.08162 (19)	0.61184 (12)	0.50674 (12)	0.0419 (5)	
C21B	1.01170 (19)	0.71283 (12)	0.35719 (11)	0.0334 (5)	
C22B	0.90840 (19)	0.70483 (12)	0.29420 (12)	0.0391 (5)	
C9A	0.80213 (16)	0.68192 (11)	−0.06451 (11)	0.0249 (4)	
C8A	0.84590 (16)	0.60628 (11)	−0.08668 (11)	0.0239 (4)	
C7A	0.83537 (16)	0.54155 (11)	−0.04116 (11)	0.0230 (4)	
C12A	0.77610 (16)	0.54502 (11)	0.02534 (11)	0.0222 (4)	
C11A	0.73357 (16)	0.62160 (11)	0.04646 (11)	0.0269 (4)	
C10A	0.74587 (16)	0.68720 (11)	0.00457 (11)	0.0264 (4)	
C13A	0.76571 (16)	0.47473 (11)	0.06661 (11)	0.0232 (4)	
C4A	0.81712 (16)	0.40392 (11)	0.04120 (11)	0.0230 (4)	
C5A	0.87533 (16)	0.40257 (11)	−0.02569 (11)	0.0224 (4)	
C6A	0.92214 (16)	0.33482 (11)	−0.05662 (11)	0.0241 (4)	
C1A	0.91606 (16)	0.26098 (11)	−0.01902 (11)	0.0256 (4)	
C2A	0.86306 (16)	0.26195 (11)	0.05052 (11)	0.0262 (4)	
C3A	0.81486 (16)	0.32957 (11)	0.07845 (11)	0.0247 (4)	
C14A	0.68673 (16)	0.47247 (11)	0.12900 (11)	0.0233 (4)	
C15A	0.56556 (17)	0.45371 (11)	0.10668 (12)	0.0292 (4)	
C16A	0.48590 (17)	0.44929 (12)	0.16068 (12)	0.0319 (5)	
C17A	0.52564 (17)	0.46464 (11)	0.23766 (12)	0.0300 (4)	
C18A	0.64616 (17)	0.48172 (11)	0.26028 (12)	0.0277 (4)	
C19A	0.72793 (16)	0.48491 (11)	0.20708 (11)	0.0230 (4)	
C20A	0.85903 (16)	0.49799 (11)	0.23319 (12)	0.0235 (4)	
C27A	0.75459 (17)	0.82365 (11)	−0.08670 (12)	0.0330 (5)	
C28A	0.62064 (18)	0.81610 (13)	−0.10645 (13)	0.0409 (5)	
C25A	0.86617 (17)	0.74497 (12)	−0.17806 (11)	0.0298 (4)	
C26A	0.77640 (19)	0.72455 (13)	−0.24777 (11)	0.0387 (5)	
C23A	0.95564 (18)	0.11495 (12)	−0.01047 (12)	0.0344 (5)	
C24A	0.84570 (18)	0.05831 (12)	−0.03624 (12)	0.0368 (5)	
C21A	0.98746 (18)	0.18428 (12)	−0.12816 (12)	0.0348 (5)	
C22A	0.88023 (19)	0.18476 (12)	−0.18812 (12)	0.0386 (5)	
C29A	1.39147 (17)	0.59375 (11)	0.38322 (11)	0.0246 (4)	
C34A	1.30914 (16)	0.61475 (11)	0.32518 (11)	0.0250 (4)	
C33A	1.21080 (16)	0.56133 (11)	0.29828 (11)	0.0252 (4)	
C32A	1.19155 (17)	0.48701 (11)	0.33185 (11)	0.0257 (4)	
C31A	1.27562 (17)	0.46701 (11)	0.39004 (11)	0.0258 (4)	
C30A	1.37478 (17)	0.51900 (11)	0.41521 (11)	0.0275 (4)	
C35A	1.50052 (18)	0.64666 (12)	0.41029 (12)	0.0306 (5)	
C36A	1.61415 (18)	0.77074 (13)	0.39752 (13)	0.0410 (5)	
C37A	1.60159 (19)	0.84407 (13)	0.35256 (13)	0.0471 (6)	
C29B	1.39195 (16)	1.09548 (11)	0.88757 (11)	0.0230 (4)	
C34B	1.30324 (16)	1.11904 (11)	0.83478 (11)	0.0247 (4)	
C33B	1.21358 (16)	1.06213 (11)	0.80066 (11)	0.0235 (4)	
C32B	1.20951 (16)	0.98077 (11)	0.82163 (11)	0.0224 (4)	
C31B	1.30135 (17)	0.95803 (11)	0.87335 (11)	0.0238 (4)	
C30B	1.39119 (16)	1.01438 (11)	0.90654 (11)	0.0252 (4)	
C35B	1.49108 (17)	1.15383 (11)	0.92221 (11)	0.0262 (4)	
C36B	1.58894 (19)	1.28594 (12)	0.91921 (13)	0.0406 (5)	
C37B	1.5856 (2)	1.35179 (14)	0.86645 (14)	0.0554 (7)	
H6BO	1.0703 (19)	1.0521 (13)	0.7266 (12)	0.052 (7)*	
H5BO	1.049 (2)	0.9436 (15)	0.7960 (14)	0.089 (9)*	
H5AO	1.029 (2)	0.4605 (14)	0.3091 (13)	0.078 (9)*	
H6AO	1.067 (2)	0.5509 (14)	0.2251 (13)	0.076 (8)*	
H4BO	1.348 (2)	0.8715 (14)	0.9351 (14)	0.081 (9)*	
H4AO	1.3091 (19)	0.3857 (13)	0.4612 (12)	0.057 (7)*	
H8B	0.8469	1.1128	0.3621	0.029*	
H11B	0.6763	1.1265	0.5899	0.031*	
H10B	0.6615	1.2321	0.5141	0.034*	
H6B	0.9676	0.8603	0.3903	0.028*	
H2B	0.8913	0.7265	0.5666	0.030*	
H3B	0.8021	0.8331	0.6183	0.029*	
H15B	0.5389	0.9587	0.5580	0.032*	
H16B	0.4125	0.9474	0.6506	0.036*	
H17B	0.4883	0.9539	0.7786	0.034*	
H18B	0.6910	0.9682	0.8115	0.032*	
H27A	0.6737	1.3542	0.3630	0.042*	
H27B	0.6630	1.3262	0.4453	0.042*	
H28A	0.5017	1.2689	0.3165	0.075*	
H28B	0.4737	1.3348	0.3798	0.075*	
H28C	0.4901	1.2441	0.3996	0.075*	
H25A	0.6927	1.2548	0.2621	0.037*	
H25B	0.7436	1.1705	0.2787	0.037*	
H26A	0.8799	1.3220	0.3029	0.054*	
H26B	0.8876	1.2571	0.2342	0.054*	
H26C	0.9304	1.2371	0.3177	0.054*	
H23A	0.9463	0.5873	0.4197	0.037*	
H23B	0.9023	0.6246	0.4952	0.037*	
H24A	1.1434	0.6135	0.4737	0.063*	
H24B	1.0743	0.5585	0.5259	0.063*	
H24C	1.1008	0.6529	0.5487	0.063*	
H21A	1.0623	0.6682	0.3492	0.040*	
H21B	1.0584	0.7641	0.3544	0.040*	
H22A	0.8532	0.6604	0.3029	0.059*	
H22B	0.9374	0.6940	0.2456	0.059*	
H22C	0.8693	0.7551	0.2943	0.059*	
H8A	0.8816	0.6000	−0.1316	0.029*	
H11A	0.6959	0.6272	0.0906	0.032*	
H10A	0.7173	0.7367	0.0209	0.032*	
H6A	0.9575	0.3369	−0.1016	0.029*	
H2A	0.8616	0.2151	0.0773	0.031*	
H3A	0.7792	0.3275	0.1233	0.030*	
H15A	0.5382	0.4441	0.0550	0.035*	
H16A	0.4057	0.4360	0.1452	0.038*	
H17A	0.4721	0.4635	0.2739	0.036*	
H18A	0.6727	0.4912	0.3121	0.033*	
H27C	0.7880	0.8677	−0.1136	0.040*	
H27D	0.7728	0.8388	−0.0323	0.040*	
H28D	0.6017	0.8054	−0.1607	0.061*	
H28E	0.5887	0.8664	−0.0900	0.061*	
H28F	0.5869	0.7717	−0.0811	0.061*	
H25C	0.9241	0.7040	−0.1767	0.036*	
H25D	0.9078	0.7978	−0.1825	0.036*	
H26D	0.7305	0.6745	−0.2419	0.058*	
H26E	0.8171	0.7179	−0.2925	0.058*	
H26F	0.7247	0.7684	−0.2531	0.058*	
H23C	0.9597	0.1285	0.0442	0.041*	
H23D	1.0252	0.0863	−0.0202	0.041*	
H24D	0.7766	0.0889	−0.0352	0.055*	
H24E	0.8410	0.0151	−0.0025	0.055*	
H24F	0.8498	0.0350	−0.0873	0.055*	
H21C	1.0256	0.1336	−0.1366	0.042*	
H21D	1.0440	0.2294	−0.1349	0.042*	
H22D	0.8188	0.1456	−0.1773	0.058*	
H22E	0.9031	0.1704	−0.2379	0.058*	
H22F	0.8513	0.2386	−0.1869	0.058*	
H34A	1.3199	0.6648	0.3042	0.030*	
H30A	1.4304	0.5042	0.4534	0.033*	
H36A	1.6819	0.7418	0.3844	0.049*	
H36B	1.6254	0.7873	0.4518	0.049*	
H37A	1.5847	0.8265	0.2992	0.071*	
H37B	1.6741	0.8786	0.3608	0.071*	
H37C	1.5379	0.8743	0.3688	0.071*	
H34B	1.3040	1.1733	0.8222	0.030*	
H30B	1.4508	0.9983	0.9414	0.030*	
H36C	1.6631	1.2599	0.9191	0.049*	
H36D	1.5822	1.3083	0.9708	0.049*	
H37D	1.5943	1.3291	0.8158	0.083*	
H37E	1.6491	1.3929	0.8830	0.083*	
H37F	1.5111	1.3762	0.8662	0.083*	

### Atomic displacement parameters (Å^2^)

**Table:**

	*U*^11^	*U*^22^	*U*^33^	*U*^12^	*U*^13^	*U*^23^
O1B	0.0235 (7)	0.0198 (6)	0.0263 (8)	0.0035 (5)	0.0056 (5)	0.0030 (5)
O2B	0.0230 (7)	0.0336 (8)	0.0277 (8)	0.0003 (6)	0.0005 (6)	0.0079 (6)
O3B	0.0199 (7)	0.0332 (7)	0.0264 (8)	−0.0033 (6)	0.0022 (6)	0.0035 (6)
O1A	0.0270 (7)	0.0206 (6)	0.0251 (8)	0.0004 (5)	0.0055 (6)	0.0029 (5)
O2A	0.0295 (7)	0.0341 (8)	0.0250 (8)	0.0019 (6)	−0.0011 (6)	0.0053 (6)
O3A	0.0216 (7)	0.0382 (8)	0.0271 (8)	−0.0027 (6)	0.0015 (6)	0.0020 (6)
O6A	0.0264 (7)	0.0280 (7)	0.0312 (9)	0.0000 (6)	−0.0048 (6)	0.0043 (6)
O5A	0.0246 (8)	0.0245 (7)	0.0494 (10)	−0.0009 (6)	−0.0070 (7)	0.0012 (6)
O4A	0.0312 (8)	0.0278 (7)	0.0373 (9)	−0.0014 (6)	−0.0038 (7)	0.0106 (6)
O7A	0.0369 (9)	0.0469 (9)	0.0520 (10)	−0.0111 (7)	−0.0197 (8)	0.0210 (8)
O8A	0.0266 (7)	0.0290 (7)	0.0375 (9)	−0.0048 (6)	−0.0042 (6)	0.0065 (6)
O6B	0.0253 (7)	0.0249 (7)	0.0335 (9)	−0.0012 (6)	−0.0085 (6)	0.0051 (6)
O5B	0.0230 (7)	0.0248 (7)	0.0372 (9)	−0.0003 (6)	−0.0022 (6)	−0.0005 (6)
O4B	0.0327 (8)	0.0239 (7)	0.0327 (9)	−0.0003 (6)	−0.0039 (6)	0.0076 (6)
O7B	0.0295 (8)	0.0345 (8)	0.0337 (9)	−0.0001 (6)	−0.0082 (6)	0.0070 (6)
O8B	0.0296 (7)	0.0244 (7)	0.0387 (9)	−0.0072 (6)	−0.0108 (6)	0.0057 (6)
N2B	0.0309 (9)	0.0271 (9)	0.0268 (10)	0.0075 (7)	0.0060 (7)	0.0045 (7)
N1B	0.0361 (9)	0.0227 (8)	0.0257 (10)	0.0074 (7)	0.0095 (7)	0.0044 (7)
N2A	0.0303 (9)	0.0240 (8)	0.0275 (10)	0.0031 (7)	0.0068 (7)	0.0063 (7)
N1A	0.0339 (9)	0.0242 (9)	0.0284 (10)	0.0057 (7)	0.0029 (8)	0.0019 (7)
C9B	0.0229 (10)	0.0238 (10)	0.0300 (13)	0.0001 (8)	0.0018 (8)	0.0042 (8)
C8B	0.0235 (10)	0.0239 (10)	0.0247 (12)	−0.0007 (8)	0.0039 (8)	0.0024 (8)
C7B	0.0186 (9)	0.0185 (9)	0.0274 (12)	−0.0001 (7)	0.0004 (8)	−0.0013 (8)
C12B	0.0202 (10)	0.0234 (10)	0.0211 (11)	−0.0010 (8)	0.0010 (8)	0.0002 (8)
C11B	0.0248 (10)	0.0278 (10)	0.0247 (12)	0.0013 (8)	0.0051 (8)	0.0005 (8)
C10B	0.0299 (11)	0.0241 (10)	0.0313 (13)	0.0050 (8)	0.0071 (9)	0.0025 (9)
C13B	0.0178 (9)	0.0241 (10)	0.0222 (11)	−0.0034 (8)	−0.0036 (8)	0.0015 (8)
C4B	0.0186 (9)	0.0210 (9)	0.0237 (12)	−0.0019 (8)	0.0009 (8)	0.0011 (8)
C5B	0.0208 (10)	0.0207 (9)	0.0222 (12)	−0.0009 (7)	−0.0020 (8)	0.0052 (8)
C6B	0.0235 (10)	0.0249 (10)	0.0235 (12)	0.0015 (8)	0.0047 (8)	0.0044 (8)
C1B	0.0219 (10)	0.0236 (10)	0.0281 (12)	0.0024 (8)	−0.0011 (8)	0.0028 (8)
C2B	0.0262 (10)	0.0232 (10)	0.0264 (12)	−0.0002 (8)	0.0027 (9)	0.0053 (8)
C3B	0.0259 (10)	0.0237 (10)	0.0227 (12)	−0.0002 (8)	0.0022 (8)	0.0029 (8)
C14B	0.0222 (10)	0.0169 (9)	0.0277 (12)	−0.0001 (7)	0.0030 (8)	0.0033 (8)
C15B	0.0240 (10)	0.0305 (11)	0.0251 (12)	−0.0014 (8)	−0.0009 (8)	0.0052 (9)
C16B	0.0205 (10)	0.0313 (11)	0.0374 (14)	−0.0017 (8)	0.0023 (9)	0.0065 (9)
C17B	0.0243 (10)	0.0328 (11)	0.0291 (13)	0.0017 (9)	0.0096 (9)	0.0046 (9)
C18B	0.0260 (11)	0.0278 (10)	0.0268 (12)	−0.0010 (8)	0.0019 (9)	0.0043 (9)
C19B	0.0203 (10)	0.0181 (9)	0.0274 (12)	−0.0012 (7)	0.0011 (8)	0.0033 (8)
C20B	0.0195 (10)	0.0211 (9)	0.0222 (12)	0.0009 (8)	0.0004 (8)	−0.0015 (8)
C27B	0.0443 (13)	0.0273 (11)	0.0377 (14)	0.0122 (10)	0.0101 (10)	0.0081 (9)
C28B	0.0410 (14)	0.0589 (16)	0.0525 (17)	0.0226 (12)	0.0041 (12)	0.0061 (13)
C25B	0.0330 (12)	0.0292 (11)	0.0316 (13)	0.0077 (9)	0.0045 (9)	0.0056 (9)
C26B	0.0402 (13)	0.0307 (11)	0.0380 (14)	0.0004 (10)	0.0082 (10)	0.0064 (10)
C23B	0.0391 (12)	0.0211 (10)	0.0335 (13)	0.0048 (9)	0.0054 (10)	0.0011 (9)
C24B	0.0508 (14)	0.0310 (12)	0.0430 (15)	0.0057 (11)	−0.0029 (11)	0.0056 (10)
C21B	0.0445 (13)	0.0272 (11)	0.0319 (13)	0.0107 (9)	0.0130 (10)	0.0042 (9)
C22B	0.0576 (15)	0.0273 (11)	0.0326 (14)	0.0036 (10)	0.0062 (11)	0.0006 (9)
C9A	0.0190 (10)	0.0260 (10)	0.0285 (12)	−0.0020 (8)	−0.0010 (8)	0.0038 (8)
C8A	0.0232 (10)	0.0266 (10)	0.0209 (11)	−0.0032 (8)	0.0023 (8)	0.0007 (8)
C7A	0.0198 (10)	0.0214 (10)	0.0266 (12)	0.0001 (8)	0.0001 (8)	−0.0009 (8)
C12A	0.0202 (10)	0.0259 (10)	0.0196 (11)	−0.0024 (8)	−0.0003 (8)	0.0020 (8)
C11A	0.0248 (10)	0.0292 (11)	0.0270 (12)	0.0013 (8)	0.0061 (8)	0.0011 (9)
C10A	0.0283 (11)	0.0234 (10)	0.0279 (12)	0.0032 (8)	0.0049 (9)	0.0015 (8)
C13A	0.0195 (9)	0.0259 (10)	0.0220 (12)	−0.0039 (8)	−0.0036 (8)	0.0012 (8)
C4A	0.0194 (10)	0.0233 (10)	0.0247 (12)	−0.0027 (8)	−0.0020 (8)	0.0028 (8)
C5A	0.0203 (10)	0.0227 (10)	0.0234 (12)	−0.0016 (8)	−0.0015 (8)	0.0041 (8)
C6A	0.0238 (10)	0.0259 (10)	0.0218 (12)	0.0000 (8)	0.0005 (8)	0.0011 (8)
C1A	0.0224 (10)	0.0243 (10)	0.0288 (12)	0.0010 (8)	−0.0025 (8)	0.0022 (8)
C2A	0.0265 (10)	0.0240 (10)	0.0272 (12)	−0.0015 (8)	−0.0013 (9)	0.0058 (8)
C3A	0.0254 (10)	0.0267 (10)	0.0211 (12)	−0.0013 (8)	0.0004 (8)	0.0023 (8)
C14A	0.0211 (10)	0.0220 (10)	0.0271 (12)	0.0004 (8)	0.0023 (8)	0.0050 (8)
C15A	0.0274 (11)	0.0330 (11)	0.0260 (12)	−0.0026 (9)	−0.0013 (9)	0.0050 (9)
C16A	0.0201 (10)	0.0348 (12)	0.0408 (14)	−0.0012 (9)	0.0022 (9)	0.0071 (10)
C17A	0.0271 (11)	0.0331 (11)	0.0315 (13)	0.0019 (9)	0.0092 (9)	0.0066 (9)
C18A	0.0323 (11)	0.0254 (10)	0.0258 (12)	0.0029 (9)	0.0040 (9)	0.0036 (8)
C19A	0.0262 (10)	0.0191 (9)	0.0234 (12)	0.0012 (8)	0.0004 (8)	0.0027 (8)
C20A	0.0229 (10)	0.0196 (10)	0.0272 (13)	0.0003 (8)	−0.0003 (9)	0.0015 (8)
C27A	0.0382 (12)	0.0241 (11)	0.0387 (14)	0.0028 (9)	0.0097 (10)	0.0074 (9)
C28A	0.0404 (13)	0.0380 (13)	0.0497 (15)	0.0147 (10)	0.0162 (11)	0.0136 (11)
C25A	0.0304 (11)	0.0270 (11)	0.0344 (13)	0.0037 (9)	0.0090 (9)	0.0082 (9)
C26A	0.0434 (13)	0.0420 (13)	0.0317 (14)	0.0103 (11)	0.0042 (10)	0.0023 (10)
C23A	0.0385 (13)	0.0273 (11)	0.0381 (14)	0.0100 (9)	0.0004 (10)	0.0057 (9)
C24A	0.0422 (13)	0.0293 (11)	0.0407 (14)	0.0063 (10)	0.0091 (11)	0.0043 (10)
C21A	0.0412 (13)	0.0285 (11)	0.0369 (14)	0.0080 (10)	0.0114 (10)	0.0027 (9)
C22A	0.0486 (14)	0.0323 (12)	0.0336 (14)	0.0002 (10)	0.0037 (11)	−0.0019 (10)
C29A	0.0245 (10)	0.0243 (10)	0.0244 (12)	0.0002 (8)	0.0025 (8)	−0.0001 (8)
C34A	0.0260 (10)	0.0225 (10)	0.0264 (12)	0.0027 (8)	0.0030 (9)	0.0010 (8)
C33A	0.0232 (10)	0.0270 (10)	0.0254 (12)	0.0054 (8)	0.0018 (8)	0.0001 (8)
C32A	0.0226 (10)	0.0211 (10)	0.0320 (13)	0.0010 (8)	0.0011 (9)	−0.0036 (8)
C31A	0.0275 (11)	0.0218 (10)	0.0286 (12)	0.0021 (8)	0.0048 (9)	0.0028 (8)
C30A	0.0258 (10)	0.0292 (11)	0.0264 (12)	0.0016 (8)	−0.0034 (9)	0.0039 (9)
C35A	0.0330 (12)	0.0283 (11)	0.0304 (13)	0.0018 (9)	0.0009 (9)	0.0068 (9)
C36A	0.0332 (12)	0.0380 (13)	0.0480 (15)	−0.0121 (10)	−0.0067 (11)	0.0081 (11)
C37A	0.0322 (12)	0.0424 (13)	0.0659 (18)	−0.0087 (10)	−0.0017 (12)	0.0191 (12)
C29B	0.0217 (10)	0.0239 (10)	0.0232 (11)	0.0010 (8)	0.0024 (8)	0.0026 (8)
C34B	0.0238 (10)	0.0214 (10)	0.0288 (12)	0.0024 (8)	0.0018 (8)	0.0026 (8)
C33B	0.0209 (10)	0.0246 (10)	0.0254 (12)	0.0042 (8)	0.0016 (8)	0.0037 (8)
C32B	0.0207 (10)	0.0224 (10)	0.0238 (12)	0.0014 (8)	0.0026 (8)	−0.0013 (8)
C31B	0.0270 (10)	0.0203 (10)	0.0247 (12)	0.0023 (8)	0.0046 (8)	0.0034 (8)
C30B	0.0230 (10)	0.0293 (10)	0.0228 (12)	0.0038 (8)	−0.0020 (8)	0.0032 (8)
C35B	0.0252 (11)	0.0242 (10)	0.0297 (13)	0.0029 (8)	0.0030 (9)	0.0038 (9)
C36B	0.0363 (13)	0.0327 (12)	0.0479 (15)	−0.0137 (10)	−0.0092 (11)	0.0056 (10)
C37B	0.0402 (14)	0.0499 (15)	0.0739 (19)	−0.0140 (12)	−0.0099 (13)	0.0287 (13)

### Geometric parameters (Å, °)

**Table:**

O1B—C7B	1.369 (2)	C21B—H21B	0.970
O1B—C5B	1.377 (2)	C22B—H22A	0.960
O2B—C20B	1.266 (2)	C22B—H22B	0.960
O3B—C20B	1.254 (2)	C22B—H22C	0.960
O1A—C7A	1.366 (2)	C9A—C8A	1.409 (2)
O1A—C5A	1.375 (2)	C9A—C10A	1.436 (2)
O2A—C20A	1.266 (2)	C8A—C7A	1.382 (2)
O3A—C20A	1.255 (2)	C8A—H8A	0.930
O6A—C33A	1.357 (2)	C7A—C12A	1.414 (2)
O6A—H6AO	0.94 (2)	C12A—C11A	1.412 (2)
O5A—C32A	1.356 (2)	C12A—C13A	1.411 (2)
O5A—H5AO	0.92 (2)	C11A—C10A	1.355 (2)
O4A—C31A	1.370 (2)	C11A—H11A	0.930
O4A—H4AO	0.92 (2)	C10A—H10A	0.930
O7A—C35A	1.216 (2)	C13A—C4A	1.398 (2)
O8A—C35A	1.336 (2)	C13A—C14A	1.495 (2)
O8A—C36A	1.455 (2)	C4A—C5A	1.413 (2)
O6B—C33B	1.353 (2)	C4A—C3A	1.425 (2)
O6B—H6BO	0.94 (2)	C5A—C6A	1.365 (2)
O5B—C32B	1.357 (2)	C6A—C1A	1.422 (2)
O5B—H5BO	0.94 (2)	C6A—H6A	0.930
O4B—C31B	1.376 (2)	C1A—C2A	1.424 (2)
O4B—H4BO	0.97 (2)	C2A—C3A	1.351 (2)
O7B—C35B	1.219 (2)	C2A—H2A	0.930
O8B—C35B	1.336 (2)	C3A—H3A	0.930
O8B—C36B	1.449 (2)	C14A—C15A	1.399 (2)
N2B—C9B	1.354 (2)	C14A—C19A	1.403 (2)
N2B—C27B	1.468 (2)	C15A—C16A	1.386 (3)
N2B—C25B	1.480 (2)	C15A—H15A	0.930
N1B—C1B	1.343 (2)	C16A—C17A	1.385 (2)
N1B—C23B	1.477 (2)	C16A—H16A	0.930
N1B—C21B	1.465 (2)	C17A—C18A	1.390 (2)
N2A—C9A	1.356 (2)	C17A—H17A	0.930
N2A—C27A	1.472 (2)	C18A—C19A	1.392 (2)
N2A—C25A	1.469 (2)	C18A—H18A	0.930
N1A—C1A	1.357 (2)	C19A—C20A	1.509 (2)
N1A—C23A	1.471 (2)	C27A—C28A	1.520 (2)
N1A—C21A	1.465 (2)	C27A—H27C	0.970
C9B—C8B	1.410 (2)	C27A—H27D	0.970
C9B—C10B	1.433 (2)	C28A—H28D	0.960
C8B—C7B	1.378 (2)	C28A—H28E	0.960
C8B—H8B	0.930	C28A—H28F	0.960
C7B—C12B	1.411 (2)	C25A—C26A	1.515 (2)
C12B—C11B	1.414 (2)	C25A—H25C	0.970
C12B—C13B	1.416 (2)	C25A—H25D	0.970
C11B—C10B	1.360 (2)	C26A—H26D	0.960
C11B—H11B	0.930	C26A—H26E	0.960
C10B—H10B	0.930	C26A—H26F	0.960
C13B—C4B	1.400 (2)	C23A—C24A	1.514 (2)
C13B—C14B	1.490 (2)	C23A—H23C	0.970
C4B—C5B	1.409 (2)	C23A—H23D	0.970
C4B—C3B	1.427 (2)	C24A—H24D	0.960
C5B—C6B	1.366 (2)	C24A—H24E	0.960
C6B—C1B	1.422 (2)	C24A—H24F	0.960
C6B—H6B	0.930	C21A—C22A	1.527 (2)
C1B—C2B	1.435 (2)	C21A—H21C	0.970
C2B—C3B	1.352 (2)	C21A—H21D	0.970
C2B—H2B	0.930	C22A—H22D	0.960
C3B—H3B	0.930	C22A—H22E	0.960
C14B—C15B	1.397 (2)	C22A—H22F	0.960
C14B—C19B	1.405 (2)	C29A—C34A	1.390 (2)
C15B—C16B	1.377 (2)	C29A—C30A	1.394 (2)
C15B—H15B	0.930	C29A—C35A	1.479 (2)
C16B—C17B	1.386 (2)	C34A—C33A	1.392 (2)
C16B—H16B	0.930	C34A—H34A	0.930
C17B—C18B	1.385 (2)	C33A—C32A	1.406 (2)
C17B—H17B	0.930	C32A—C31A	1.398 (2)
C18B—C19B	1.385 (2)	C31A—C30A	1.380 (2)
C18B—H18B	0.930	C30A—H30A	0.930
C19B—C20B	1.506 (2)	C36A—C37A	1.492 (3)
C27B—C28B	1.521 (2)	C36A—H36A	0.970
C27B—H27A	0.970	C36A—H36B	0.970
C27B—H27B	0.970	C37A—H37A	0.960
C28B—H28A	0.960	C37A—H37B	0.960
C28B—H28B	0.960	C37A—H37C	0.960
C28B—H28C	0.960	C29B—C34B	1.392 (2)
C25B—C26B	1.512 (2)	C29B—C30B	1.392 (2)
C25B—H25A	0.970	C29B—C35B	1.477 (2)
C25B—H25B	0.970	C34B—C33B	1.390 (2)
C26B—H26A	0.960	C34B—H34B	0.930
C26B—H26B	0.960	C33B—C32B	1.407 (2)
C26B—H26C	0.960	C32B—C31B	1.400 (2)
C23B—C24B	1.516 (2)	C31B—C30B	1.381 (2)
C23B—H23A	0.970	C30B—H30B	0.930
C23B—H23B	0.970	C36B—C37B	1.474 (3)
C24B—H24A	0.960	C36B—H36C	0.970
C24B—H24B	0.960	C36B—H36D	0.970
C24B—H24C	0.960	C37B—H37D	0.960
C21B—C22B	1.520 (2)	C37B—H37E	0.960
C21B—H21A	0.970	C37B—H37F	0.960
			
O2B···O5B	2.5636 (18)	O4A···O7A^i^	2.779 (2)
O3B···O6B	2.5811 (17)	O7A···O4A^i^	2.779 (2)
O2A···O5A	2.5690 (19)	O6B···O3B	2.5811 (17)
O3A···O6A	2.6303 (17)	O5B···O2B	2.5636 (18)
O6A···O3A	2.6303 (17)	O4B···O7B^ii^	2.7957 (19)
O5A···O2A	2.5690 (19)	O7B···O4B^ii^	2.7957 (19)
			
C7B—O1B—C5B	120.28 (14)	C13A—C4A—C3A	124.15 (18)
C7A—O1A—C5A	120.36 (14)	C5A—C4A—C3A	115.50 (16)
C33A—O6A—H6AO	116.1 (15)	O1A—C5A—C4A	120.19 (16)
C32A—O5A—H5AO	108.2 (14)	O1A—C5A—C6A	115.71 (17)
C31A—O4A—H4AO	112.7 (13)	C4A—C5A—C6A	124.05 (17)
C35A—O8A—C36A	116.30 (15)	C5A—C6A—C1A	119.04 (18)
C33B—O6B—H6BO	117.3 (13)	C5A—C6A—H6A	120.5
C32B—O5B—H5BO	108.4 (14)	C1A—C6A—H6A	120.5
C31B—O4B—H4BO	108.8 (14)	N1A—C1A—C6A	120.83 (17)
C35B—O8B—C36B	116.75 (14)	N1A—C1A—C2A	121.22 (16)
C9B—N2B—C27B	122.96 (16)	C6A—C1A—C2A	117.94 (16)
C9B—N2B—C25B	121.36 (15)	C1A—C2A—C3A	121.38 (17)
C27B—N2B—C25B	115.26 (15)	C1A—C2A—H2A	119.3
C1B—N1B—C23B	123.73 (16)	C3A—C2A—H2A	119.3
C1B—N1B—C21B	121.62 (15)	C4A—C3A—C2A	121.98 (18)
C23B—N1B—C21B	114.30 (15)	C4A—C3A—H3A	119.0
C9A—N2A—C27A	121.28 (16)	C2A—C3A—H3A	119.0
C9A—N2A—C25A	122.30 (15)	C13A—C14A—C15A	116.74 (16)
C27A—N2A—C25A	115.95 (15)	C13A—C14A—C19A	123.83 (15)
C1A—N1A—C23A	121.87 (16)	C15A—C14A—C19A	119.38 (18)
C1A—N1A—C21A	121.48 (15)	C14A—C15A—C16A	120.74 (18)
C23A—N1A—C21A	115.77 (15)	C14A—C15A—H15A	119.6
N2B—C9B—C8B	121.31 (17)	C16A—C15A—H15A	119.6
N2B—C9B—C10B	121.14 (17)	C15A—C16A—C17A	120.03 (17)
C8B—C9B—C10B	117.48 (17)	C15A—C16A—H16A	120.0
C9B—C8B—C7B	119.55 (18)	C17A—C16A—H16A	120.0
C9B—C8B—H8B	120.2	C16A—C17A—C18A	119.49 (19)
C7B—C8B—H8B	120.2	C16A—C17A—H17A	120.3
O1B—C7B—C8B	115.67 (17)	C18A—C17A—H17A	120.3
O1B—C7B—C12B	120.71 (16)	C17A—C18A—C19A	121.39 (18)
C8B—C7B—C12B	123.58 (17)	C17A—C18A—H18A	119.3
C7B—C12B—C11B	115.51 (17)	C19A—C18A—H18A	119.3
C7B—C12B—C13B	119.58 (16)	C14A—C19A—C18A	118.90 (16)
C11B—C12B—C13B	124.90 (18)	C14A—C19A—C20A	120.64 (17)
C12B—C11B—C10B	122.45 (18)	C18A—C19A—C20A	120.40 (17)
C12B—C11B—H11B	118.8	O2A—C20A—O3A	125.32 (16)
C10B—C11B—H11B	118.8	O2A—C20A—C19A	116.83 (17)
C9B—C10B—C11B	120.88 (18)	O3A—C20A—C19A	117.84 (17)
C9B—C10B—H10B	119.6	N2A—C27A—C28A	113.88 (14)
C11B—C10B—H10B	119.5	N2A—C27A—H27C	108.8
C12B—C13B—C4B	118.65 (17)	N2A—C27A—H27D	108.8
C12B—C13B—C14B	119.22 (16)	C28A—C27A—H27C	108.8
C4B—C13B—C14B	121.43 (16)	C28A—C27A—H27D	108.8
C13B—C4B—C5B	120.14 (17)	H27C—C27A—H27D	107.7
C13B—C4B—C3B	124.36 (18)	C27A—C28A—H28D	109.5
C5B—C4B—C3B	115.49 (16)	C27A—C28A—H28E	109.5
O1B—C5B—C4B	120.50 (16)	C27A—C28A—H28F	109.5
O1B—C5B—C6B	115.06 (17)	H28D—C28A—H28E	109.5
C4B—C5B—C6B	124.31 (17)	H28D—C28A—H28F	109.5
C5B—C6B—C1B	119.11 (18)	H28E—C28A—H28F	109.5
C5B—C6B—H6B	120.4	N2A—C25A—C26A	112.96 (15)
C1B—C6B—H6B	120.5	N2A—C25A—H25C	109.0
N1B—C1B—C6B	121.08 (17)	N2A—C25A—H25D	109.0
N1B—C1B—C2B	121.41 (16)	C26A—C25A—H25C	109.0
C6B—C1B—C2B	117.48 (16)	C26A—C25A—H25D	109.0
C1B—C2B—C3B	121.37 (17)	H25C—C25A—H25D	107.8
C1B—C2B—H2B	119.3	C25A—C26A—H26D	109.5
C3B—C2B—H2B	119.3	C25A—C26A—H26E	109.5
C4B—C3B—C2B	121.89 (18)	C25A—C26A—H26F	109.5
C4B—C3B—H3B	119.1	H26D—C26A—H26E	109.5
C2B—C3B—H3B	119.1	H26D—C26A—H26F	109.5
C13B—C14B—C15B	117.01 (16)	H26E—C26A—H26F	109.5
C13B—C14B—C19B	124.05 (16)	N1A—C23A—C24A	113.03 (16)
C15B—C14B—C19B	118.90 (17)	N1A—C23A—H23C	109.0
C14B—C15B—C16B	121.18 (17)	N1A—C23A—H23D	109.0
C14B—C15B—H15B	119.4	C24A—C23A—H23C	109.0
C16B—C15B—H15B	119.4	C24A—C23A—H23D	109.0
C15B—C16B—C17B	119.98 (17)	H23C—C23A—H23D	107.8
C15B—C16B—H16B	120.0	C23A—C24A—H24D	109.5
C17B—C16B—H16B	120.0	C23A—C24A—H24E	109.5
C16B—C17B—C18B	119.23 (19)	C23A—C24A—H24F	109.5
C16B—C17B—H17B	120.4	H24D—C24A—H24E	109.5
C18B—C17B—H17B	120.4	H24D—C24A—H24F	109.5
C17B—C18B—C19B	121.70 (18)	H24E—C24A—H24F	109.5
C17B—C18B—H18B	119.1	N1A—C21A—C22A	113.73 (17)
C19B—C18B—H18B	119.2	N1A—C21A—H21C	108.8
C14B—C19B—C18B	118.97 (16)	N1A—C21A—H21D	108.8
C14B—C19B—C20B	120.97 (17)	C22A—C21A—H21C	108.8
C18B—C19B—C20B	120.06 (16)	C22A—C21A—H21D	108.8
O2B—C20B—O3B	124.95 (16)	H21C—C21A—H21D	107.7
O2B—C20B—C19B	117.26 (16)	C21A—C22A—H22D	109.5
O3B—C20B—C19B	117.78 (16)	C21A—C22A—H22E	109.5
N2B—C27B—C28B	112.09 (15)	C21A—C22A—H22F	109.5
N2B—C27B—H27A	109.2	H22D—C22A—H22E	109.5
N2B—C27B—H27B	109.2	H22D—C22A—H22F	109.5
C28B—C27B—H27A	109.2	H22E—C22A—H22F	109.5
C28B—C27B—H27B	109.2	C34A—C29A—C30A	119.76 (16)
H27A—C27B—H27B	107.9	C34A—C29A—C35A	122.05 (17)
C27B—C28B—H28A	109.5	C30A—C29A—C35A	118.13 (16)
C27B—C28B—H28B	109.5	C29A—C34A—C33A	120.45 (17)
C27B—C28B—H28C	109.5	C29A—C34A—H34A	119.8
H28A—C28B—H28B	109.5	C33A—C34A—H34A	119.8
H28A—C28B—H28C	109.5	O6A—C33A—C34A	116.93 (16)
H28B—C28B—H28C	109.5	O6A—C33A—C32A	123.01 (15)
N2B—C25B—C26B	113.12 (14)	C34A—C33A—C32A	120.06 (16)
N2B—C25B—H25A	109.0	O5A—C32A—C33A	122.47 (16)
N2B—C25B—H25B	109.0	O5A—C32A—C31A	118.98 (16)
C26B—C25B—H25A	109.0	C33A—C32A—C31A	118.50 (16)
C26B—C25B—H25B	109.0	O4A—C31A—C32A	116.68 (15)
H25A—C25B—H25B	107.8	O4A—C31A—C30A	121.98 (16)
C25B—C26B—H26A	109.5	C32A—C31A—C30A	121.34 (17)
C25B—C26B—H26B	109.5	C29A—C30A—C31A	119.83 (17)
C25B—C26B—H26C	109.5	C29A—C30A—H30A	120.1
H26A—C26B—H26B	109.5	C31A—C30A—H30A	120.1
H26A—C26B—H26C	109.5	O7A—C35A—O8A	122.74 (17)
H26B—C26B—H26C	109.5	O7A—C35A—C29A	124.11 (18)
N1B—C23B—C24B	111.32 (15)	O8A—C35A—C29A	113.15 (16)
N1B—C23B—H23A	109.4	O8A—C36A—C37A	106.78 (16)
N1B—C23B—H23B	109.4	O8A—C36A—H36A	110.4
C24B—C23B—H23A	109.4	O8A—C36A—H36B	110.4
C24B—C23B—H23B	109.4	C37A—C36A—H36A	110.4
H23A—C23B—H23B	108.0	C37A—C36A—H36B	110.4
C23B—C24B—H24A	109.5	H36A—C36A—H36B	108.6
C23B—C24B—H24B	109.5	C36A—C37A—H37A	109.5
C23B—C24B—H24C	109.5	C36A—C37A—H37B	109.5
H24A—C24B—H24B	109.5	C36A—C37A—H37C	109.5
H24A—C24B—H24C	109.5	H37A—C37A—H37B	109.5
H24B—C24B—H24C	109.5	H37A—C37A—H37C	109.5
N1B—C21B—C22B	112.00 (17)	H37B—C37A—H37C	109.5
N1B—C21B—H21A	109.2	C34B—C29B—C30B	119.96 (15)
N1B—C21B—H21B	109.2	C34B—C29B—C35B	121.61 (16)
C22B—C21B—H21A	109.2	C30B—C29B—C35B	118.38 (16)
C22B—C21B—H21B	109.2	C29B—C34B—C33B	120.49 (16)
H21A—C21B—H21B	107.9	C29B—C34B—H34B	119.8
C21B—C22B—H22A	109.5	C33B—C34B—H34B	119.7
C21B—C22B—H22B	109.5	O6B—C33B—C34B	116.78 (16)
C21B—C22B—H22C	109.5	O6B—C33B—C32B	123.26 (15)
H22A—C22B—H22B	109.5	C34B—C33B—C32B	119.95 (16)
H22A—C22B—H22C	109.5	O5B—C32B—C33B	122.60 (16)
H22B—C22B—H22C	109.5	O5B—C32B—C31B	118.89 (16)
N2A—C9A—C8A	122.07 (17)	C33B—C32B—C31B	118.43 (15)
N2A—C9A—C10A	120.35 (16)	O4B—C31B—C32B	116.59 (15)
C8A—C9A—C10A	117.58 (17)	O4B—C31B—C30B	121.92 (16)
C9A—C8A—C7A	119.43 (18)	C32B—C31B—C30B	121.48 (17)
C9A—C8A—H8A	120.3	C29B—C30B—C31B	119.58 (16)
C7A—C8A—H8A	120.3	C29B—C30B—H30B	120.2
O1A—C7A—C8A	115.54 (17)	C31B—C30B—H30B	120.2
O1A—C7A—C12A	120.98 (16)	O7B—C35B—O8B	122.68 (15)
C8A—C7A—C12A	123.45 (17)	O7B—C35B—C29B	124.46 (17)
C7A—C12A—C11A	115.86 (17)	O8B—C35B—C29B	112.86 (15)
C7A—C12A—C13A	119.47 (16)	O8B—C36B—C37B	107.23 (16)
C11A—C12A—C13A	124.65 (18)	O8B—C36B—H36C	110.3
C12A—C11A—C10A	122.18 (18)	O8B—C36B—H36D	110.3
C12A—C11A—H11A	118.9	C37B—C36B—H36C	110.3
C10A—C11A—H11A	118.9	C37B—C36B—H36D	110.3
C9A—C10A—C11A	121.38 (17)	H36C—C36B—H36D	108.5
C9A—C10A—H10A	119.3	C36B—C37B—H37D	109.5
C11A—C10A—H10A	119.3	C36B—C37B—H37E	109.5
C12A—C13A—C4A	118.63 (17)	C36B—C37B—H37F	109.5
C12A—C13A—C14A	120.20 (16)	H37D—C37B—H37E	109.5
C4A—C13A—C14A	120.58 (16)	H37D—C37B—H37F	109.5
C13A—C4A—C5A	120.35 (17)	H37E—C37B—H37F	109.5
			
C7B—O1B—C5B—C4B	−3.1 (2)	C14B—C19B—C20B—O3B	22.2 (2)
C7B—O1B—C5B—C6B	172.93 (14)	C18B—C19B—C20B—O2B	23.2 (2)
C5B—O1B—C7B—C8B	−172.94 (14)	C18B—C19B—C20B—O3B	−157.74 (17)
C5B—O1B—C7B—C12B	4.6 (2)	N2A—C9A—C8A—C7A	178.73 (16)
C7A—O1A—C5A—C4A	−0.4 (2)	N2A—C9A—C10A—C11A	179.06 (16)
C7A—O1A—C5A—C6A	177.32 (14)	C8A—C9A—C10A—C11A	−1.0 (2)
C5A—O1A—C7A—C8A	−178.16 (14)	C10A—C9A—C8A—C7A	−1.2 (2)
C5A—O1A—C7A—C12A	0.2 (2)	C9A—C8A—C7A—O1A	−177.84 (14)
C35A—O8A—C36A—C37A	177.21 (17)	C9A—C8A—C7A—C12A	3.9 (2)
C36A—O8A—C35A—O7A	4.5 (2)	O1A—C7A—C12A—C11A	177.77 (15)
C36A—O8A—C35A—C29A	−175.36 (16)	O1A—C7A—C12A—C13A	−0.7 (2)
C35B—O8B—C36B—C37B	165.84 (17)	C8A—C7A—C12A—C11A	−4.0 (2)
C36B—O8B—C35B—O7B	3.9 (2)	C8A—C7A—C12A—C13A	177.50 (16)
C36B—O8B—C35B—C29B	−175.40 (16)	C7A—C12A—C11A—C10A	1.7 (2)
C9B—N2B—C27B—C28B	−87.6 (2)	C7A—C12A—C13A—C4A	1.4 (2)
C27B—N2B—C9B—C8B	178.66 (15)	C7A—C12A—C13A—C14A	−169.81 (15)
C27B—N2B—C9B—C10B	1.6 (2)	C11A—C12A—C13A—C4A	−176.87 (16)
C9B—N2B—C25B—C26B	−89.2 (2)	C11A—C12A—C13A—C14A	11.9 (2)
C25B—N2B—C9B—C8B	6.5 (2)	C13A—C12A—C11A—C10A	−179.93 (16)
C25B—N2B—C9B—C10B	−170.61 (15)	C12A—C11A—C10A—C9A	0.7 (2)
C27B—N2B—C25B—C26B	98.01 (18)	C12A—C13A—C4A—C5A	−1.7 (2)
C25B—N2B—C27B—C28B	85.0 (2)	C12A—C13A—C4A—C3A	178.79 (16)
C1B—N1B—C23B—C24B	−96.8 (2)	C12A—C13A—C14A—C15A	81.0 (2)
C23B—N1B—C1B—C6B	−175.11 (16)	C12A—C13A—C14A—C19A	−101.5 (2)
C23B—N1B—C1B—C2B	2.8 (2)	C4A—C13A—C14A—C15A	−90.1 (2)
C1B—N1B—C21B—C22B	−79.9 (2)	C4A—C13A—C14A—C19A	87.4 (2)
C21B—N1B—C1B—C6B	−2.3 (2)	C14A—C13A—C4A—C5A	169.51 (15)
C21B—N1B—C1B—C2B	175.63 (16)	C14A—C13A—C4A—C3A	−10.0 (2)
C23B—N1B—C21B—C22B	93.53 (19)	C13A—C4A—C5A—O1A	1.2 (2)
C21B—N1B—C23B—C24B	89.91 (19)	C13A—C4A—C5A—C6A	−176.33 (16)
C9A—N2A—C27A—C28A	−73.2 (2)	C13A—C4A—C3A—C2A	178.16 (17)
C27A—N2A—C9A—C8A	173.33 (15)	C5A—C4A—C3A—C2A	−1.4 (2)
C27A—N2A—C9A—C10A	−6.7 (2)	C3A—C4A—C5A—O1A	−179.26 (14)
C9A—N2A—C25A—C26A	93.2 (2)	C3A—C4A—C5A—C6A	3.2 (2)
C25A—N2A—C9A—C8A	1.5 (2)	O1A—C5A—C6A—C1A	−179.54 (14)
C25A—N2A—C9A—C10A	−178.48 (15)	C4A—C5A—C6A—C1A	−1.9 (2)
C27A—N2A—C25A—C26A	−79.03 (19)	C5A—C6A—C1A—N1A	177.70 (16)
C25A—N2A—C27A—C28A	99.11 (19)	C5A—C6A—C1A—C2A	−1.3 (2)
C1A—N1A—C23A—C24A	93.3 (2)	N1A—C1A—C2A—C3A	−175.92 (16)
C23A—N1A—C1A—C6A	178.26 (15)	C6A—C1A—C2A—C3A	3.0 (2)
C23A—N1A—C1A—C2A	−2.8 (2)	C1A—C2A—C3A—C4A	−1.7 (2)
C1A—N1A—C21A—C22A	−66.2 (2)	C13A—C14A—C15A—C16A	179.23 (17)
C21A—N1A—C1A—C6A	−13.0 (2)	C13A—C14A—C19A—C18A	179.76 (17)
C21A—N1A—C1A—C2A	165.94 (16)	C13A—C14A—C19A—C20A	−2.8 (2)
C23A—N1A—C21A—C22A	103.19 (18)	C15A—C14A—C19A—C18A	−2.7 (2)
C21A—N1A—C23A—C24A	−76.1 (2)	C15A—C14A—C19A—C20A	174.71 (16)
N2B—C9B—C8B—C7B	−170.64 (16)	C19A—C14A—C15A—C16A	1.6 (2)
N2B—C9B—C10B—C11B	170.33 (16)	C14A—C15A—C16A—C17A	0.9 (2)
C8B—C9B—C10B—C11B	−6.9 (2)	C15A—C16A—C17A—C18A	−2.1 (2)
C10B—C9B—C8B—C7B	6.5 (2)	C16A—C17A—C18A—C19A	0.9 (2)
C9B—C8B—C7B—O1B	176.91 (14)	C17A—C18A—C19A—C14A	1.5 (2)
C9B—C8B—C7B—C12B	−0.5 (2)	C17A—C18A—C19A—C20A	−175.93 (16)
O1B—C7B—C12B—C11B	177.48 (14)	C14A—C19A—C20A—O2A	−163.72 (16)
O1B—C7B—C12B—C13B	−3.7 (2)	C14A—C19A—C20A—O3A	15.0 (2)
C8B—C7B—C12B—C11B	−5.2 (2)	C18A—C19A—C20A—O2A	13.7 (2)
C8B—C7B—C12B—C13B	173.66 (16)	C18A—C19A—C20A—O3A	−167.57 (16)
C7B—C12B—C11B—C10B	4.9 (2)	C34A—C29A—C30A—C31A	−1.2 (2)
C7B—C12B—C13B—C4B	1.2 (2)	C30A—C29A—C34A—C33A	−0.6 (2)
C7B—C12B—C13B—C14B	−169.33 (15)	C34A—C29A—C35A—O7A	−174.4 (2)
C11B—C12B—C13B—C4B	179.95 (14)	C34A—C29A—C35A—O8A	5.5 (2)
C11B—C12B—C13B—C14B	9.4 (2)	C35A—C29A—C34A—C33A	176.44 (18)
C13B—C12B—C11B—C10B	−173.88 (17)	C30A—C29A—C35A—O7A	2.7 (3)
C12B—C11B—C10B—C9B	1.0 (2)	C30A—C29A—C35A—O8A	−177.43 (17)
C12B—C13B—C4B—C5B	0.3 (2)	C35A—C29A—C30A—C31A	−178.35 (18)
C12B—C13B—C4B—C3B	−178.75 (16)	C29A—C34A—C33A—O6A	−177.54 (17)
C12B—C13B—C14B—C15B	69.8 (2)	C29A—C34A—C33A—C32A	2.6 (2)
C12B—C13B—C14B—C19B	−112.6 (2)	O6A—C33A—C32A—O5A	0.0 (2)
C4B—C13B—C14B—C15B	−100.5 (2)	O6A—C33A—C32A—C31A	177.44 (17)
C4B—C13B—C14B—C19B	77.1 (2)	C34A—C33A—C32A—O5A	179.93 (17)
C14B—C13B—C4B—C5B	170.59 (15)	C34A—C33A—C32A—C31A	−2.7 (2)
C14B—C13B—C4B—C3B	−8.4 (2)	O5A—C32A—C31A—O4A	−1.6 (2)
C13B—C4B—C5B—O1B	0.6 (2)	O5A—C32A—C31A—C30A	178.38 (18)
C13B—C4B—C5B—C6B	−174.98 (16)	C33A—C32A—C31A—O4A	−179.07 (17)
C13B—C4B—C3B—C2B	176.06 (16)	C33A—C32A—C31A—C30A	0.9 (2)
C5B—C4B—C3B—C2B	−3.0 (2)	O4A—C31A—C30A—C29A	−179.02 (17)
C3B—C4B—C5B—O1B	179.72 (14)	C32A—C31A—C30A—C29A	1.0 (3)
C3B—C4B—C5B—C6B	4.1 (2)	C34B—C29B—C30B—C31B	−0.7 (2)
O1B—C5B—C6B—C1B	−175.98 (14)	C30B—C29B—C34B—C33B	−0.0 (2)
C4B—C5B—C6B—C1B	−0.2 (2)	C34B—C29B—C35B—O7B	175.68 (19)
C5B—C6B—C1B—N1B	173.16 (16)	C34B—C29B—C35B—O8B	−5.0 (2)
C5B—C6B—C1B—C2B	−4.8 (2)	C35B—C29B—C34B—C33B	177.35 (18)
N1B—C1B—C2B—C3B	−172.05 (16)	C30B—C29B—C35B—O7B	−6.9 (3)
C6B—C1B—C2B—C3B	5.9 (2)	C30B—C29B—C35B—O8B	172.40 (17)
C1B—C2B—C3B—C4B	−2.0 (2)	C35B—C29B—C30B—C31B	−178.20 (18)
C13B—C14B—C15B—C16B	179.90 (16)	C29B—C34B—C33B—O6B	−177.61 (17)
C13B—C14B—C19B—C18B	−179.28 (16)	C29B—C34B—C33B—C32B	2.5 (2)
C13B—C14B—C19B—C20B	0.8 (2)	O6B—C33B—C32B—O5B	−0.6 (3)
C15B—C14B—C19B—C18B	−1.7 (2)	O6B—C33B—C32B—C31B	176.04 (18)
C15B—C14B—C19B—C20B	178.41 (16)	C34B—C33B—C32B—O5B	179.29 (18)
C19B—C14B—C15B—C16B	2.1 (2)	C34B—C33B—C32B—C31B	−4.0 (2)
C14B—C15B—C16B—C17B	−0.8 (2)	O5B—C32B—C31B—O4B	1.3 (2)
C15B—C16B—C17B—C18B	−1.0 (2)	O5B—C32B—C31B—C30B	−179.88 (18)
C16B—C17B—C18B—C19B	1.4 (2)	C33B—C32B—C31B—O4B	−175.53 (17)
C17B—C18B—C19B—C14B	−0.1 (2)	C33B—C32B—C31B—C30B	3.3 (2)
C17B—C18B—C19B—C20B	179.85 (16)	O4B—C31B—C30B—C29B	177.84 (18)
C14B—C19B—C20B—O2B	−156.93 (16)	C32B—C31B—C30B—C29B	−0.9 (3)

Symmetry codes: (i) −*x*+3, −*y*+1, −*z*+1; (ii) −*x*+3, −*y*+2, −*z*+2.

### Hydrogen-bond geometry (Å, °)

**Table:**

*D*—H···*A*	*D*—H	H···*A*	*D*···*A*	*D*—H···*A*
O6A—H6AO···O2A	0.94 (2)	2.72 (2)	3.4413 (18)	133.8 (18)
O6A—H6AO···O3A	0.94 (2)	1.72 (2)	2.6303 (17)	162 (2)
O6A—H6AO···O5A	0.94 (2)	2.53 (2)	2.8733 (19)	102.2 (17)
O5A—H5AO···O2A	0.92 (2)	1.66 (2)	2.5690 (19)	167 (2)
O5A—H5AO···O3A	0.92 (2)	2.62 (2)	3.1884 (18)	120.4 (18)
O4A—H4AO···O7A^i^	0.92 (2)	1.86 (2)	2.779 (2)	178.8 (10)
O6B—H6BO···O2B	0.94 (2)	2.72 (2)	3.4156 (18)	131.4 (16)
O6B—H6BO···O3B	0.94 (2)	1.68 (2)	2.5811 (17)	159 (2)
O6B—H6BO···O5B	0.94 (2)	2.55 (2)	2.8799 (19)	101.0 (15)
O5B—H5BO···O2B	0.94 (2)	1.64 (2)	2.5636 (18)	166 (2)
O5B—H5BO···O3B	0.94 (2)	2.66 (2)	3.2473 (18)	121.5 (19)
O4B—H4BO···O7B^ii^	0.97 (2)	1.83 (2)	2.7957 (19)	175 (2)

Symmetry codes: (i) −*x*+3, −*y*+1, −*z*+1; (ii) −*x*+3, −*y*+2, −*z*+2.
